# P1492: A NEW POTENTIAL AND PREVALENT SIDE EFFECT OF FERRIC CARBOXYMALTOSE: METHEMOGLOBINEMIA. A CASE‐CONTROL STUDY.

**DOI:** 10.1097/01.HS9.0000972848.82517.3f

**Published:** 2023-08-08

**Authors:** Muhammet Özbilen, Şeyda Tuba Savrun, Ali Aygün, Yasemin Kaya

**Affiliations:** ^1^ Ordu University School of Medicine Internal Medicine Ordu Turkey; ^2^ Ankara Diskapi Yildirim Beyazit Training and Research Hospital Emergency Medicine Ankara Turkey; ^3^ Ordu University School of Medicine Emergency Medicine Ordu Turkey; ^4^ Ordu University School of Medicine Internal Medicine Ordu Turkey

Abstract Topic: 29. Iron metabolism, deficiency and overload

**Background:** With the new generation of intravenous iron drugs, it is now possible to administer higher doses of iron at once. However, there are no published studies on the effects of old or new generations of intravenous iron replacement therapies on blood gas parameters.

**Aims:** We recently reported the first case of methemoglobinemia due to ferric carboxymaltose (FCM). In addition to this single case reported in the literature, we wondered whether methemoglobinemia has been observed following FCM over time. In order to investigate the presence of this new potential side effect, we aimed to retrospectively review blood gas data after FCM infusions.

**Methods:** The study sample comprised 833 individuals who were scheduled to get FCM therapy at the Ordu University Training and Research Hospital in 2021. This sample included 35 patients whose venous blood gas was measured following FCM infusion. There were a total of thirty‐one individuals for whom the number of FCM vials administered was documented. One vial of the commercial FCM product (Ferinject®) contains 500 mg of iron in total. According to the amount of FCM administered, there were 25 patients who received double vials and 6 patients who received single vials. To compare the groups who got FCM to those who did not get FCM, an additional 25 patients with unremarkable venous blood gases were added in the study. The patients’ blood gas, hemogram, c‐reactive protein, and iron parameters were analyzed using one‐way analysis of variance, post hoc, and correlation tests.

**Results:** In all but two of the patients who had FCM infusion, the amount of methemoglobin was more than 1%. It was observed that two patients without methemoglobinemia received only one vial. The median values of methemoglobin were 1.65% (0.7‐2.2%), 2.03% (1.1‐3.2%) and 0.60% (0.1‐1.0%) for single vial, two vials and control group patients, respectively. The significant differences (Chi square=39.2, p<0.001, df =2) were found among the three groups. In the post hoc test, both FCM‐receiving groups significantly differed from the control group (p=0.01 and p<0.001). Nevertheless, there was no significant difference between the groups that received FCM (p=0.17). In addition, a significant (p<0.01) and moderate correlation was seen between methemoglobin levels and hemoglobin, mean corpuscular volume, and transferrin saturations, as estimated by the correlation coefficients (r): ‐0.460, ‐0.491, and ‐0.567. No clinical symptom details, if any, were documented in patient files following FCM infusion.

**Summary/Conclusion:** In this study, the finding of mild methemoglobinemia following ferric carboxymaltose infusion is remarkable. All biochemical and clinical aspects of this side effect must be investigated in depth. On the other hand, the clinical importance of methemoglobinemia due to FCM remains a mystery. We intend to conduct a prospective study involving a larger number of patients in order to better comprehend and describe all these new developments.

**Keywords:** Anemia, Iron deficiency, Side effects, Hemoglobin

